# Multisensory attention training for treatment of tinnitus

**DOI:** 10.1038/srep10802

**Published:** 2015-05-28

**Authors:** Spiegel D. P., Linford T., Thompson B., Petoe M. A., Kobayashi K., Stinear C. M., Searchfield G. D.

**Affiliations:** 1 Section of Audiology, School of Population Health, The University of Auckland, 261 Morrin Road, Glenn Innes, Auckland, New Zealand; 2 Centre for Brain Research, The University of Auckland, 85 Park Road, Grafton, Auckland, New Zealand; 3 Department of Optometry and Vision Science, The University of Auckland, 85 Park Road, Grafton, Auckland, New Zealand; 4 The Bionics Institute of Australia, 384-388 Albert Street, Melbourne, Australia; 5 McGill Vision Research, Department of Ophthalmology, McGill University, Montreal, Canada

## Abstract

Tinnitus is the conscious perception of sound with no physical sound source. Some models of tinnitus pathophysiology suggest that networks associated with attention, memory, distress and multisensory experience are involved in tinnitus perception. The aim of this study was to evaluate whether a multisensory attention training paradigm which used audio, visual, and somatosensory stimulation would reduce tinnitus. Eighteen participants with predominantly unilateral chronic tinnitus were randomized between two groups receiving 20 daily sessions of either integration (attempting to reduce salience to tinnitus by binding with multisensory stimuli) or attention diversion (multisensory stimuli opposite side to tinnitus) training. The training resulted in small but statistically significant reductions in Tinnitus Functional Index and Tinnitus Severity Numeric Scale scores and improved attentional abilities. No statistically significant improvements in tinnitus were found between the training groups. This study demonstrated that a short period of multisensory attention training reduced unilateral tinnitus, but directing attention toward or away from the tinnitus side did not differentiate this effect.

Tinnitus is the conscious awareness of sound without an external driving sound. It is believed to arise from an injury to the peripheral auditory system^1^ resulting in deafferentation; the lack of normal sensory input is thought to trigger a cascade of neuroplastic changes within the subcortical and cortical auditory pathways^2^. There is often little direct relationship between the subjectively reported severity of tinnitus and the psychoacoustic matches such as loudness and sensation level^3^,^4^ suggesting the involvement of processes related to attention, emotion, and memory in tinnitus pathophysiology^5^. Support for this assumption is the observation showing that unilateral tinnitus is associated with a marked attention shift towards the tinnitus ear^6^. Participants with a unilateral tinnitus were able to detect target sounds more accurately in the tinnitus ear than the opposite ear but this did not occur in a simulated tinnitus group^6^. Further support comes from functional imaging studies. Although the evidence is not univocal^7^ and many of the methods used are still under development, the majority of recent studies using electroencephalography (EEG)^8^ resting state functional magnetic resonance imaging (fMRI)^9^,^10^, and positron emission tomography (PET) scans^11^ have shown altered brain connectivity in tinnitus patients. As an example, Vanneste *et al*^8^. showed increased EEG gamma connectivity between the left auditory cortex, left insula, left parahippocampus, and the right dorsolateral prefrontal cortex arguing that these connections may be linked to maintaining consciousness and the enduring attention towards the tinnitus sound. PET in tinnitus patients has revealed increased resting state activity in limbic, frontal and parietal regions and a positive correlation between tinnitus duration and the activity in the right inferior frontal, right ventro-medial prefrontal, and the right posterior cingulate cortices^11^.

Multisensory interactions appear to be a ubiquitous property of information processing within the central nervous system. The evidence comes both from behavioural and physiological studies as well as well-known phenomena such as the McGurk effect^12^. For example, an irrelevant auditory cue can modulate the response to a visual target^13^ and vice versa^14^. Similarly, irrelevant auditory or visual cues can affect reaction times for tactile targets^15^. These behavioural data are supported by several neuroimaging and neuroanatomical studies that have identified a convergence of inputs from multiple senses at several cortical and subcortical loci^16^,^17^ including the auditory cortex^18^. There is evidence indicating involvement of multisensory interactions in tinnitus^19^,^20^,^21^,^22^,^23^,^24^,^25^. Animal noise studies showed increased bimodal interaction at the dorsal cochlear nucleus^19^,^26^. In humans, various somatosensory manipulations, in particular around the head region, may evoke tinnitus or alter its psychoacoustic characteristics such as pitch and/or loudness^20^. For example, jaw movements may elicit an increase in tinnitus loudness^21^ that is associated with increased activation of the auditory brain areas^22^. Another intriguing, although rare, example is a form of tinnitus that can be modulated upon peripheral eye gaze^23^,^24^,^25^. These changes in tinnitus perception have been associated with reduced inhibition and increased activity within the central auditory system^24^. However, at least one study suggests that multisensory training (learning to play music) has less effect than unisensory training (listening music) in a notched music paradigm^27^.

The proposed involvement of attention and multisensory connection in tinnitus may be related to auditory scene analysis; a process in which the auditory system identifies and differentiates auditory objects and segregates relevant information from background noise^28^,^29^. It has been suggested that auditory scene analysis involves assigning different sound features to a source which is, under normal circumstances, represented by a physical object. Relevant sounds are attended to while irrelevant sounds become habituated. Tinnitus is unusual because, in addition to the strong emotional response it elicits, it does not have a localizable external source and therefore violates the rules of auditory scene analysis^28^. This may result in attentional resources being directed towards the tinnitus sound in order to validate its ecological relevance and purpose^28^ which prevents habituation^30^. A potential dissociation between auditory cortex and visual cortex in tinnitus patients has also recently been demonstrated in MRI, raising further questions to the role of sensory integration and tinnitus^31^.

Considering the research described, an intervention targeting attention and the multisensory interactions may represent a promising tool for the treatment of tinnitus. Furthermore, it has been shown that results of multisensory perceptual learning are superior to unisensory training^32^,^33^. Here, we introduce a novel approach to tinnitus treatment which involves combining attention training with multisensory (auditory, visual and somatosensory) stimulation. We hypothesize that multisensory attentional training will reduce tinnitus within multiple tinnitus severity domains as measured with the Tinnitus Functional Index (TFI)^34^. Two possible approaches emerge in light of the proposed role of auditory scene analysis in the pathophysiology of tinnitus^28^,^35^. Multisensory stimulation could be used to divert attention away from tinnitus (an *attention diversion approach*) to reduce its salience, or to integrate the sound of tinnitus with other sensory modalities (an *integration approach*). Integration of the tinnitus sound with other senses could provide a “source” for the tinnitus, possibly allowing for habituation. Putatively, both approaches have the potential to relieve the attentional load towards tinnitus and reduce subjective tinnitus annoyance^28^.

The primary aim of this study was to assess whether multisensory attention training would reduce the impact of tinnitus (as measured by the Tinnitus Functional Index (TFI)^34^ on participants. Secondary aims were to examine the effect of the multisensory training on attentional abilities and whether attention training type (diversion or integration) had a differential effect on tinnitus.

## Methods

### Participants

Eighteen participants (7 females, 11 males, mean age = 59.1 years ± 9.6 SD) were recruited from the Tinnitus database at The University of Auckland (Table 1). All participants had constant, subjective, predominantly unilateral tinnitus without any active external or middle ear pathology. They had no worse than 80 dB sensorineural hearing loss, and had normal or corrected-to-normal near vision (binocular visual acuity at 40 cm not worse than 0.3 logMAR). Participant’s eligibility was assessed at an initial audiological examination consisting of otoscopy, audiometry, a subjective computer-based tinnitus assessment, and the TFI questionnaire. The detailed methodology of the audiometry examination and tinnitus assessment are described in supplementary methods. The sample size to detect the 13 point difference in TFI was estimated considering previous TFI data from our laboratory^36^ and assuming the power of 0.8. All procedures were in accordance with the tenets of the Declaration of Helsinki and were approved by the Health and Disability Ethics Committees (HDECs), New Zealand. Informed consent was obtained from all participants prior to data collection.

### Multisensory attention training

**Figure 1:**
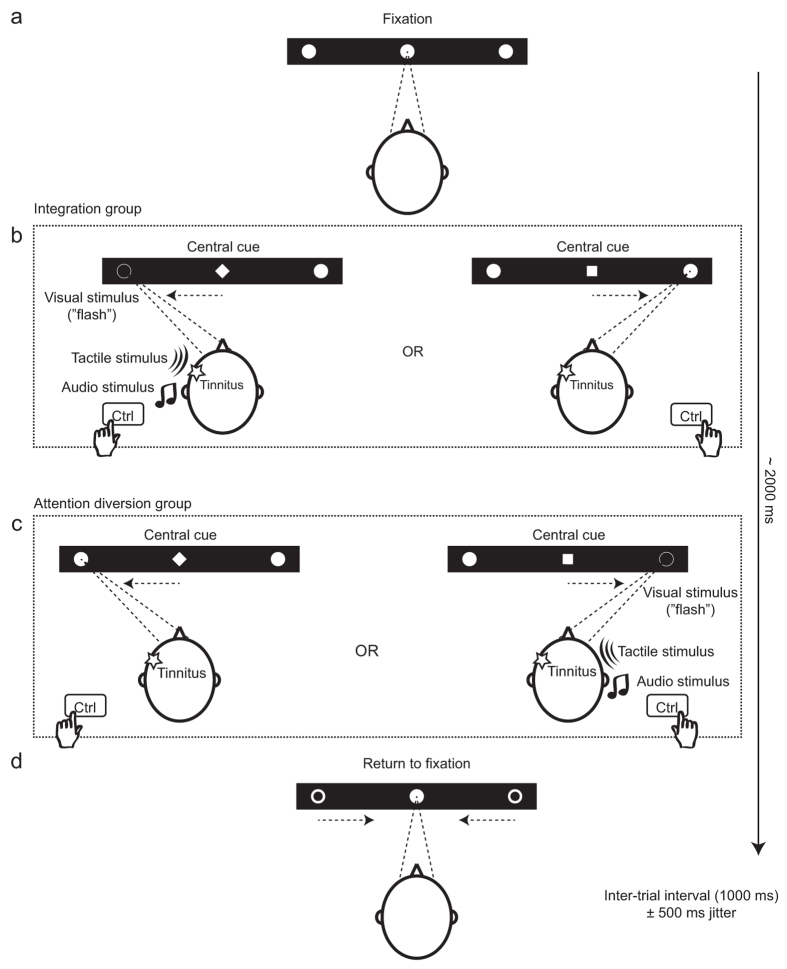
Multisensory attention training; an example of a participant with left-dominant tinnitus. In this case, the change of the centre dot into a diamond always indicates a saccade to the left dot and a change into a square indicates a saccade to the right dot. Each trial started with fixation of the centre dot (**a**). In the integration group (**b**), the diamond cue is accompanied by a flash of the left dot, an auditory stimulus delivered to the left ear and vibration administered on the left temple (tinnitus side). The square cue is not accompanied with lateralized stimuli. In the attention diversion group (**c**), the diamond is presented alone, whereas the square is associated with flashing of the right dot and tactile and auditory stimuli presented to the right side (non-tinnitus side). Participants were also instructed to press the left or right Ctrl-key on a computer keyboard according to the direction of their saccade. Each trial terminated when the lateralized dots became hollow which cued the participant to re-fixate on the centre dot for the next trial.

The multisensory training relied on repeated completion of a simple visual task that was combined with auditory and tactile stimulation (Fig. 1). At the beginning of each trial, participants were presented with three horizontally aligned white dots on a black background. Participants fixated on the centre dot which changed from a dot to a diamond or a square to indicate that a saccadic eye movement should be made to the left or right target dot. For half of the participants, the diamond signalled a leftward saccade and the square a rightward saccade across all sessions. This was reversed for the other half of the participants. In addition, participants were required to press a key on the left or right of a computer keyboard (one of the two Ctrl keys) which corresponded to the direction of their saccade. Participants were encouraged to achieve the best possible reaction time. One second after the centre dot changed shape, the target dots became hollow indicating that the participant should return their fixation to the centre dot (Fig. 1). Participants were encouraged to perform this task as accurately and quickly as possible. Simultaneously with the central cue (a diamond or square), an additional, lateralized visual cue was provided whereby the relevant target dot was flashed. This was accompanied by simultaneous, lateralized auditory and tactile cues. The auditory stimulus was a monaural pure tone presented for 120 ms. The tone’s pitch was measured and adjusted (“calibrated”) on a day-to-day basis to match the subjective tinnitus pitch. The tactile stimulus comprised of a brief vibration (120 ms) delivered to the participant’s temple. Each trial lasted approximately 2000 ms, depending on each participant’s reaction time, and an inter-trial interval of 1000 ms was jittered by ± up to 500 ms.

Participants were assigned to one of two experimental groups, the *integration group* and the *attention diversion group*. In the integration group, all three lateralized stimuli (auditory, visual and tactile) were presented when the central cue indicated a saccade towards the tinnitus side. No cues were presented when the central cue indicated a saccade towards the non-tinnitus side. This mapping was reversed for the diversion group (Fig. 1). The two groups were matched for age (independent sample *t* = 0.19, *p*  = 0.85), tinnitus duration (*t *= 0.37, *p *= 0.72), baseline TFI score (*t *= 0.285, *p *= 0.77), gender (Pearson *Χ*^*2*^ = 0.234, *p *= 0.63), and central cue directionality (diamond means to look left vs. diamond means to look right).

The treatment consisted of 20 unsupervised daily sessions (approximately 20 – 30 minutes per session) completed by the participant at home. In each session participants completed 160 trials with equal numbers of left- and right-oriented trials divided into four blocks. To increase the motivation, the average reaction time was presented after each block of 40 trials. Prior to each daily session, participants performed a rapid computer based self-assessment of their tinnitus using a custom software utility within the training program. These data were used to track the effect of treatment on tinnitus perception and adjust the auditory stimulus in pitch and loudness for the training session (see below for more details).

The training software was programmed using Psychtoolbox (version 3.0.10)^37^,^38^ for Matlab R2012b (MathWorks) and delivered using Dell Latitude E6400 laptops (2.26 GHz, 4 GB RAM). Auditory stimuli were presented via calibrated MB770G/B Apple earbuds. Tactile stimulation was delivered via Arduino LilyPads (Little Bird Electronics, Lilypad-vibe-board, DEV-11008) vibrating patches sewn into a HALO® Rhythm head band. The patches were positioned at the left and right temples.

### Primary outcome measure TFI

The primary outcome measure was the 25 item version of TFI^34^ with 11 point numeric scale (from 0 or 0% indicating no problem to 10 or 100% indicating a very big problem). This version has recently been validated for test-retest reliability and internal consistency in New Zealand^39^ and the UK^40^. The TFI was evaluated before (Pre) after (Post) and three weeks after (Post 3w) the training. In addition, it was completed at the initial audiology assessment (Baseline) in order to provide a measure of test-retest reliability within our cohort and to match the two experimental groups for tinnitus severity. The Baseline and Pre visits were scheduled on average 7.8 ± 6.7 SD weeks apart from one another.

### Secondary outcome measures

The secondary outcome measures employed in the study were selected in order to further evaluate the effects of the treatment on participants’ tinnitus and their attentional resources. Secondary outcome measures consisted of other standardized questionnaires evaluating tinnitus, mental state, and a variety of attentional measures.

### Questionnaires

All participants completed the Tinnitus Handicap Inventory (THI)^41^ and Tinnitus Severity Numeric Scale (TSNS)^42^. The Depression, Anxiety and Stress Scale (DASS)^43^ questionnaire was administered to detect any changes in the emotional state of the participants. These questionnaires were completed before (Pre), directly after (Post), and 3 weeks after (Post 3w) the multisensory training.

### Comprehensive attention battery (CAB)

The CAB (NeuropsychWorks, Inc.) is a computer-based test battery which provides an objective assessment of attention^44^. Previous studies from our group have shown that attention-based perceptual training in tinnitus had the greatest effect on a subset of CAB measures, specifically the Discriminate Reaction Time (DRT) and Auditory-Visual Multiprocessing Tests (AVMT)^35^. Therefore these tests were employed in the current study in their visual, audio and mixed sub-versions. The number of hits and hit reaction times were measured before (Pre) and after (Post) the multisensory training. Closer details about these procedures can be found in the supplementary materials.

### Eye tracking

Eye tracking was used to assess the effects of the multisensory training on visual attention^45^. During eye tracking, participants completed a visual task that was identical to the multisensory training paradigm (Fig. 1) except that no additional lateralized visual, tactile, and auditory stimuli were presented. In these trials, participants responded only by making saccadic eye movements to the dots presented to the left and right of the central dot. Each dot subtended 1.07° and the distance between the centre and each of the side dots was 12° of visual angle. The participants were seated 32 cm from the screen with the head secured in a headrest. Where necessary, an appropriate refractive correction was worn.

Saccadic latency and percent of correct saccades were assessed before (Pre) and after (Post) the multisensory training. Saccadic latency was defined as the time from the presentation of the central cue (diamond or square) to the time at which the saccade began. A saccade was defined as an eye movement that exceeded 3° in magnitude and 30°/sec in speed. Saccades with latency shorter than 80 ms were excluded from the analysis as anticipatory saccades^46^. Accuracy was assessed using the horizontal eye excursion in degrees at the time of the saccade offset which was defined as the point at which saccade velocity dropped below 30°/sec. Peak velocity was measured between saccade onset and offset. Each eye movement trace was inspected to ensure that our custom MatLab routine had identified the saccades correctly. Saccades corrupted by an eye blink and trials with a loss of tracking were excluded from analysis (5.9% of all trials).

The visual stimuli were programmed using MatLab installed on an Intel Xeon 2 GHz, 3 GB RAM desktop and presented on a Dell E771p monitor (resolution 1024 × 768, refresh rate 75 Hz). The eye tracking data were recorded using a binocular 400 Hz ViewPoint EyeTracker® (Arrington Research).

### Daily tinnitus assessment and training calibration

Prior to each daily session of multisensory training, an inbuilt routine allowing for subjective tinnitus assessment was run by each individual. Participants were first asked to identify a tone best matching their tinnitus pitch. Subsequently, they were instructed to match the intensity level of a sound at the tinnitus pitch to the perceived loudness of their tinnitus. Finally, tinnitus was localized along the x, y, and z axes. Pitch, loudness and location were estimated once using a method of adjustment with a visual analogue sliding scale. These data allowed for the auditory stimulus to be matched to the tinnitus sound during training. The loudness of the tone was set by the participant to a “comfortable level”.

### Statistical analysis

As the Pre values for the main outcome measure TFI were not normally distributed (Shapiro-Wilk test *p* < 0.05), we adopted non-parametric statistics in this study. Friedman Test with a factor of Time (Pre, Post, Post 3w) was conducted to evaluate the overall effect of the training (attention diversion and integration groups’ data pooled) for the TFI as well as for secondary questionnaire-based outcome measures TSNS, THI and DASS. The Wilcoxon signed ranks test was adopted to compare Pre vs. Post and Post vs. Post 3w, where applicable. The secondary measures CAB (number of hits and reaction time of hits separately for each test) and eye tracking (saccadic latency and percent of correct saccades separately for tinnitus dominant and non-dominant side), and tinnitus characteristics (pitch and loudness) were also assessed using the Wilcoxon signed rank test comparing the Pre and Post values.

In order to examine potential mechanisms of the training, we conducted secondary analyses. These consisted of Spearman’s correlations and Wilcoxon signed rank tests comparing the Pre and Post values separately for each treatment sub-group, and Mann–Whitney U tests comparing the Pre-to-Post changes between the groups (attention diversion vs. integration) in measures that had shown an overall significant difference. Alpha level of p < 0.05 was adopted for all analyses.

## Results

All participants finished the course of training and reported no adverse effects. Due to technical difficulties, one participant missed 2 days of training.

### Primary outcome measure – Tinnitus Functional Index

**Figure 2:**
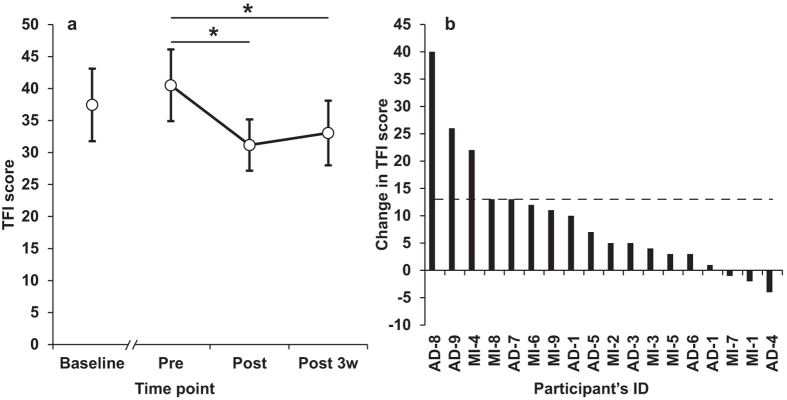
The effect of multisensory training on the TFI. Panel (**a**) shows mean TFI scores from the initial auditory examination (Baseline), before (Pre), after (Post) and three weeks after (Post 3w) the multisensory training. ** p < 0.01, * = p < 0.05. Error bars represent ± SEM. Panel (**b**) shows individual’s change in TFI from Pre to Post training. The dashed line represents the proposed level of clinically meaningful change in TFI^34^.

The TFI scores suggest that most of the participants benefited from the multisensory training (Fig. 2). After 20 days of treatment, 5/16 (31.3%) participants showed 13 point or greater improvement, 3/16 (18.7%) patients showed an improvement between 10 and 12 TFI points, the remaining 5 participants showed a change of less than 7 points. At the group level, there was an overall decrease in the TFI scores over time (*χ*^*2*^_2_ = 12.343, p = 0.02); the mean reduction post treatment was 9.3 points ± 2.6 SEM (*Z* = -3.246, *p* = 0.001). The change in TFI score was approaching a positive correlation with the duration of tinnitus (Spearman’s *ρ* = 0.434, *p* = 0.072) but did not correlate with participants’ age (Spearman’s *ρ* = 0.294, p = 0.236). The TFI scores from the initial audiology examination (Baseline) did not significantly differ from those recorded directly before training (Pre) (*Z* = -0.939, *p* = 0.348) indicating that the reported improvements were likely attributable to the period of multisensory training rather than normal fluctuations in tinnitus.

### Secondary outcome measures

#### Questionnaires

**Figure 3:**
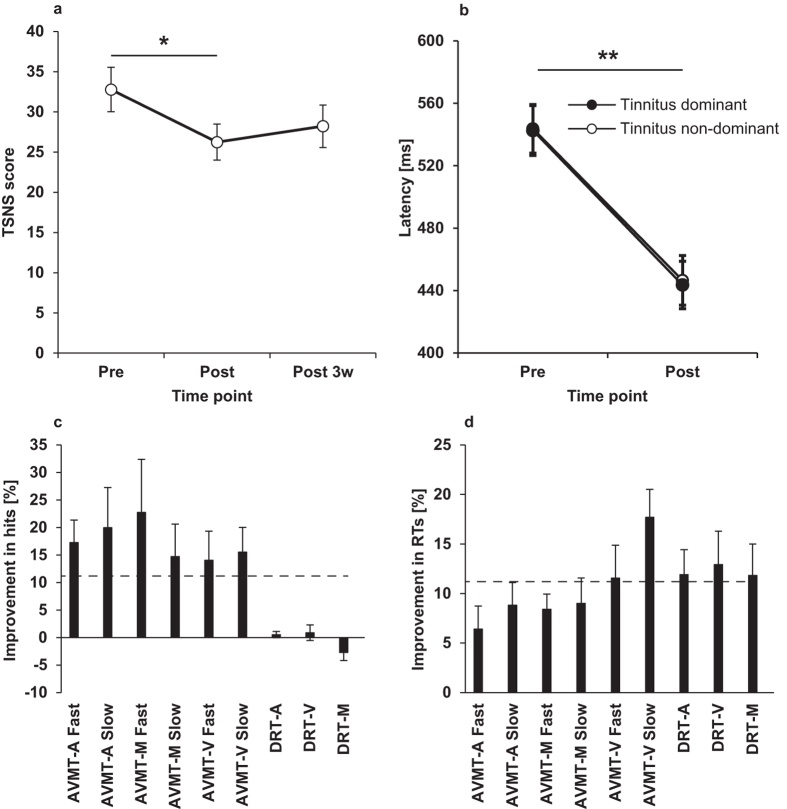
The effect of multisensory treatment on secondary outcome measures. Panel (**a**) shows mean TSNS scores before (Pre), after (Post) and three weeks after (Post 3w) the multisensory training. Panel (**b**) shows the mean saccadic latency of correctly initiated saccades towards tinnitus dominant (white circles) and non-dominant (black circles) sides. Panels (**c**) and (**d**) show the mean % change in hits and reaction time (RT) after the multisensory treatment. The dashed line represents the magnitude of procedural learning on the CAB^34^. Error bars represent +/- SEM. * p < 0.05, ** p < 0.01. AVMT-A=Auditory-Visual Multiprocessing Tests – Auditory sub-type, AVMT-M = Auditory-Visual Multiprocessing Tests – Mixed sub-type, AVMT-V=Auditory-Visual Multiprocessing Tests – Visual sub-type, DRT-A=Discriminate Reaction Time – Auditory sub-type, DRT-M=Discriminate Reaction Time – Visual sub-type, DRT-M = Discriminate Reaction Time – Mixed sub-type. Error bars represent +/- SEM. * p < 0.05, ** p < 0.01.

Wilcoxon signed ranks test conducted on TSNS scores revealed a significant decrease over time (Z = -2.484, p = 0.013) with an average reduction of 6.5 points ± 2.1 SEM (Fig. 3). There was a positive correlation between the changes in the TFI and TSNS scores (Spearman’s *ρ* = 0.776, *p* < 0.01) indicating good agreement between these two measures. No change over the course of training was associated with the THI and DASS scores.

### Comprehensive Attention Battery

Criteria of 11.2% minimum change in CAB scores were applied on the basis of procedural learning or practice effects with this test battery^36^. The analysis of the CAB data (Fig. 3) indicated that participants’ attentional abilities improved after the multisensory training as indicated by a significant increase in the number of hits in 6 of 9 CAB tests (*p* < 0.05). On average, these improvements were above learning effect for all Auditory-Visual Multiprocessing Tests but not Discriminate Reaction Time measures (Fig. 3c). In addition, there was a decrease in hit reaction time in all CAB tests which was above the learning effect in all Discriminate Reaction Time measures and 2 of 6 Auditory-Visual Multiprocessing Tests measures (Fig. 3d).

### Eye tracking

The eye tracking data of two participants were not included in the analysis due to unreliable eye tracking.

**Figure 4:**
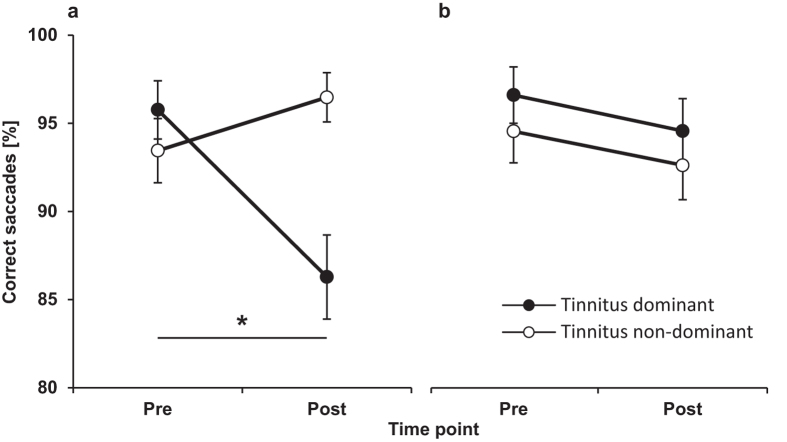
The effect of multisensory training on saccade error rates. The percentage of correct saccades is shown before (Pre) and after (Post) the multisensory training for the integration. (**a**) and attention diversion groups (**b**). Filled circles represent saccades directed towards the tinnitus side. Open circles represent saccades towards the non-tinnitus side. Error bars represent ± SEM. * indicates p < 0.05.

No differences were found for the different measures of saccadic eye movements between the tinnitus and non-tinnitus side before the training. The multisensory training resulted in significantly shorter latencies on both tinnitus dominant (*Z* = -3.413, *p* = 0.001) and non-dominant side (*Z = -*3.434*, p = *0.001). The analysis of percentage of correctly initiated saccades (Fig. 4) did not reveal any treatment-related effect on the tinnitus non-dominant side (*Z* = -0.103, *p* = 0.92) bud did significantly decrease the percent of correct saccades towards the tinnitus side (*Z = -2.608*, *p* = 0.009). Saccade velocity and accuracy were not reliably affected by training.

### Secondary analyses

Both subtypes of treatment were similarly efficient as revealed by Mann–Whitney U test comparing the Pre-Post changes in TFI between the two treatment subgroups (p > 0.05).

The decrease in percentage of correctly initiated saccades towards the tinnitus side was significant only for the integration (*Z* = 2.524, *p* = 0.012) but not for the attention diversion group (*Z* = -0.734, *p* = 0.463). A comparison of this effect between the two groups was approaching significance *U = 14.5*, *p* = 0.071.

### Tinnitus characteristics

**Figure 5:**
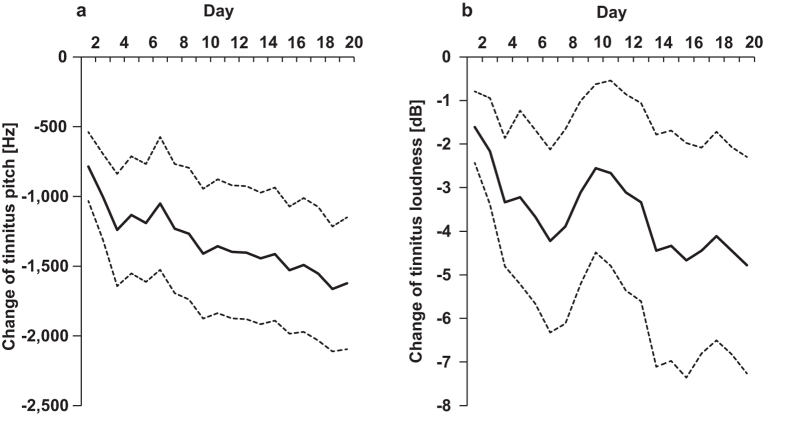
The effect of multisensory training on tinnitus characteristics. The solid lines in panels (**a**) and (**b**) represent a mean change (pooled for both groups) in tinnitus pitch and loudness, respectively, as a function of days of training. The dotted lines represent a ± 1 SEM range.

The data obtained from the daily calibration of the auditory stimuli presented during the training game showed that the mean tinnitus pitch and the sensation level (dB from hearing threshold at the pitch) were 59944 ± 748 Hz and 11.11 ± 1.87 dB (mean ± SEM), respectively at the first day of the training. There was a marked overall decrease in tinnitus pitch (*Z* = -2.95, *p* = 0.003) over the course of the training and the largest change occurred during the first five days (Fig. 5a). On average, tinnitus loudness also tended to drop over the course of treatment by an average of 5 dB. This trend approached significance (*Z* = 1.787, *p* = 0.074) (Fig. 5b). The daily calibrated data also indicated that larger changes in tinnitus pitch (Pre vs. Post) may be associated with decreased tinnitus loudness. The lack of statistical significance of this correlation (Spearman’s *ρ* = -0.463, *p* = 0.053) is driven by one participant.

## Discussion

In this study we have evaluated the effects of a novel multisensory attention training in the management of tinnitus. We have shown that a group of 18 participants improved in TFI and TSNS scores after a course of 20 days of this training. The training also led to an improvement in auditory, visual and, audio-visual attentional skills as indicated by the CAB and eye tracking measures. Our findings are in agreement with previous data showing that attentional training can improve symptoms of tinnitus^35^,^36^ and attentional abilities in people with tinnitus^35^.

The design of the task was motivated by the following requirements. The task should 1. Allow for a presentation of simple visual, auditory and tactile stimulation. 2. Allow for lateralization of the stimulus presentation, 3. Be based on an established paradigm allowing for measurement of horizontal saccadic eye movements. Such task arguably requires far transfer which may be difficult to achieve, particularly, in naïve observers^47^. In order to avoid counselling to affect the outcome of the training, participants were naïve to the rational of the task in the present study. It is therefore possible that participants might benefit from having the rational of the task explained; as understanding of the underlying principles and assumptions of training may enhance far transfer^47^.

A further aim of the current study was to compare the effectiveness of two different multisensory training approaches. The attention diversion approach has been used in previous studies aiming to divert attention away from the tinnitus sound^35^,^36^. The integration approach, however, is a novel strategy designed to integrate the tinnitus sound with visual and somatosensory stimuli. The aim of the integration approach was to reduce the conflict between the phantom auditory perception and the lack of input from other sensory modalities which has been suggested as a possible source of salience for the tinnitus sound^11^,^35^,^48^. The results indicate that both approaches are equally effective as measured with the TFI and the CAB. This result raises a concern whether laterality of tinnitus and/or stimuli matters in this particular task. Our data cannot directly answer this question. Previous studies showed that a cognitively demanding task irrelevant to tinnitus can temporarily decrease tinnitus^49^, although other results imply that tasks relevant to tinnitus may be more efficient^36^. Future studies including participants without strictly lateralized tinnitus and/or involving non-lateralized tasks are warranted.

The present data showed that the change of TFI scores was not correlated with the change in any of the attentional measures provided by the CAB. This finding implies that mechanisms other than attention were involved in the effects of the training. Participants were challenged to achieve the best possible reaction time and were presented four times with their average score during each training session. This may have provided another competing source of limbic and autonomic system activation whereby the improvement in the reaction time acted as a positive reinforcement which superseded the negative reinforcement originating from tinnitus. It has been shown that autonomic reactions occur at a subconscious level^50^. It is therefore possible that the multisensory training reduced the negative emotions associated with tinnitus independently from performance on the CAB tests that reflect conscious top-down processes^51^. Considering the above, one may also query the key role of the multisensory approach in our study. In other words, it may be possible that the same task performed only within one sensory modality may deliver comparable treatment effects. A study breaking down the individual sensory modalities and comparing their effects alone to the multisensory approach would shed more light on this question.

Lastly, we were interested whether altered attention in tinnitus affects saccadic eye movements. Cuny *et al*^6^. found that unilateral tinnitus is associated with an attentional shift towards the tinnitus side. In light of the results of their study, one would expect that the saccadic measures, error rate, latency and velocity would be imbalanced in favour of the tinnitus side^45^. Our eye tracking data, however, revealed no baseline difference between the tinnitus and non-tinnitus side in any of the saccadic measures. This finding indicates that chronic unilateral tinnitus does not affect performance on a visual attention task relying on a purely oculomotor response. This is in agreement with recent evidence showing a lack of a left-right saccadic asymmetry in unilateral tinnitus participants^52^. Interestingly, in the integration (but not diversion) group, we revealed a decreased number of correct saccades directed towards tinnitus side. This suggests that participants in this group relied more on the auditory cues during the training that was not present during the saccadic measurements. We speculate that this may reflect the integration of the tinnitus sound with the auditory, visual and/or somatosensory modalities; however, the exact neural mechanisms remain to be elucidated. Our data also revealed a task-specific decrease in saccade latency which was comparable for both training approaches. This result is not surprising as the saccadic task was part of the training paradigm that was performed for 20 consecutive days.

This study does not suggest that multisensory treatment would be more efficacious than other existing therapies. However, the overall improvement in the tinnitus questionnaires (approximately 20%) was achieved in considerably shorter time that in other studies. For example, tinnitus retraining therapy has shown 20% - 33% improvement in questionnaires and visual analogue scale in one year^53^, listening to notched music is associated with 20% - 30% decrease in tinnitus loudness in 6 to 12 months^54^, and cognitive behavioural thereby can decrease tinnitus loudness by 16% and annoyance by 37% in 6 weeks^55^. Participants self-reported modest differences in their tinnitus which were consistent with the improvement in the questionnaires and psychoacoustic measures performed each day before the training session. Interestingly, these data show a relatively large decrease in tinnitus pitch after the first day of the training (786.5 ± 234 Hz). We cannot rule out that this observation may be—at least in part—driven by procedural learning. Participants were exposed to the procedure of tinnitus pitch (and loudness) matching at the initial audiology examination as well as the study admission appointment. It is therefore possible that the pitch change after the first day may reflect a combination of the multisensory training and gained confidence in their tinnitus assessment.

In summary, there is a growing body of evidence that attention training may be an effective therapeutic approach for a variety of cortically based disorders such as amblyopia^56^,^57^, stroke^58^, and tinnitus^35^,^36^. Our preliminary results indicate that multisensory attention training may be a viable therapeutic approach for the management of tinnitus. Although the primary outcome measure (TFI) does not directly address the question whether the training acts upon improvement in emotional state or by changing tinnitus percept, the psychoacoustic data suggest the latter. Although our primary outcome measure showed a statistically significant change with time, the degree of change was modest, and its clinical relevance remains to be determined. A limitation of this study is that it did not include a placebo group. We did, however, administer the TFI at two visits before the training (Baseline and Pre) separated on average by eight weeks. If anything, the TFI scores were larger at the later visit. Studies directed towards understanding the neural correlates of the training effects are warranted, as are randomised controlled trials, before this training can be advocated as a tinnitus therapy.

## Additional Information

**How to cite this article**: Spiegel, D. P. *et al*. Multisensory attention training for treatment of tinnitus. *Sci. Rep.* **5**, 10802; doi: 10.1038/srep10802 (2015).

## Figures and Tables

**Table 1:** Participant details. MI = Multisensory Integration Group, AD = Attention Diversion Group, M = Male, F = Female. Tinnitus sensation levels are expressed as dB from hearing threshold at the tinnitus pitch.

**Group/ID**	**Age [years]**	**Gender**	**Tinnitus Laterality**	**Tinnitus Duration [months]**	**TFI Score**	**Tinnitus Sensation Level [dB]**	**Tinnitus Pitch [Hz]**
**MI-1**	65	M	Left	11	23	18	6,000
**MI-2**	59	F	Left	2	30	21	7,560
**MI-3**	71	F	Left	25	18	2	7,128
**MI-4**	46	M	Right	25	29	12	12,502
**MI-5**	57	M	Left	27	57	2	10,078
**MI-6**	62	F	Right	8	30	18	8,001
**MI-7**	63	F	Left	8	39	5	8,977
**MI-8**	44	M	Left	23	27	15	3,562
**MI-9**	61	M	Left	11	99	20	210
**AD-1**	50	M	Right	5	21	13	3,367
**AD-1**	75	F	Left	23	26	−2	6,001
**AD-3**	68	M	Left	25	41	−4	5,346
**AD-4**	63	F	Left	1	22	17	794
**AD-5**	70	F	Right	18	15	13	3,564
**AD-6**	54	M	Left	5	35	9	3,000
**AD-7**	52	M	Right	31	46	21	7,997
**AD-8**	63	M	Right	13	21	11	5,346
**AD-9**	41	M	Right	3	95	6	7,560
